# Influenza vaccination coverage: findings from immunization information systems

**DOI:** 10.1186/1471-2431-7-28

**Published:** 2007-07-27

**Authors:** Laura A Zimmerman, Diana L Bartlett, Kyle S Enger, Kimiko Gosney, Warren G Williams

**Affiliations:** 1 Immunization Services Division, National Center for Immunization and Respiratory Diseases, Centers for Disease Control and Prevention, 1600 Clifton Rd., NE, MSE-62, Atlanta, Georgia, 30333, USA; 2 Michigan Department of Community Health Division of Immunization, 5th floor, Capitol View Bldg., 201 Townsend St., PO Box 30195, Lansing, MI 48909, USA; 3 Arizona State Department of Health Services 150 N. 18th Ave., Suite 120 Phoenix AZ, 85007, USA

## Abstract

**Background:**

Beginning with the 2004–05 influenza season, the Advisory Committee on Immunization Practices (ACIP) strengthened their existing encouragement that children aged 6–23 months receive influenza vaccination by creating a formal recommendation.

**Methods:**

Well-functioning sentinel project immunization information systems (IIS) in Arizona (AIIS) and Michigan (MIIS) were used to calculate vaccination coverage among children aged 6–23 months during the 2004–05 influenza season. We calculated 2 measures of vaccination coverage: a) receipt of 1 or more doses of influenza vaccine September 2004-March 2005 and b) receipt of 2 or more doses (ie, fully vaccinated). We compared the dose administration distribution among children needing 1 and 2 doses and by provider type. Coverage by age and timeliness of vaccine doses entered into the IIS were also analyzed.

**Results:**

Influenza vaccination coverage levels among children were 30% and 27% in AIIS and MIIS, respectively, for receipt of 1 or more doses; 13% and 11% of children, respectively, were fully vaccinated. Peaks in dose administration among children needing 1 and 2 doses were similar. There were differences in vaccine administration between public and private providers. Coverage was higher among younger children and over 75% of all influenza vaccine doses were entered into the IIS within 30 days after receipt of vaccine.

**Conclusion:**

Though almost 1/3 of children received 1 or more doses of vaccine in 2 IIS sentinel projects during the first season of the new recommendation, emphasis needs to be placed on increasing the proportion of children fully vaccinated. IIS data can be used for timely monitoring of vaccination coverage assessments.

## Background

Influenza causes significant morbidity among children. In the United States, rates of influenza infection are highest among children, and those aged 6–23 months are at substantially increased risk for influenza-related hospitalizations [1]. The increased rates of hospitalizations are comparable with rates for other groups considered to be at high risk for influenza-related complications [2].

Beginning in 2002, the Advisory Committee on Immunization Practices (ACIP) encouraged the vaccination of all children aged 6–23 months with influenza vaccine. Subsequently, beginning with the 2004–05 influenza season, ACIP changed the encouragement to a recommendation that all children aged 6–23 months receive influenza vaccine annually [3]. Among previously unvaccinated children <9 years, 2 doses administered at least 1 month apart are needed for satisfactory antibody response.

Traditionally, influenza vaccination coverage assessments have been conducted using data from large national surveys, such as the National Immunization Survey (NIS) and the National Health Interview Survey (NHIS). Although these surveys provide nationally representative results, they require the use of sophisticated statistical and survey techniques that are labor and cost intensive [4]. Furthermore, these surveys are limited in providing timely coverage assessments, which are needed in many situations (eg, to provide immediate information for vaccine distribution planning during vaccine shortages and to estimate population-based vaccine-induced immunity during outbreaks).

Immunization information systems (IIS) are confidential, computerized systems that maintain vaccine administration information and have other complex capabilities (eg, vaccine management, adverse event reporting, and linkages with electronic data sources) [5]. These systems are useful in programmatic and clinical assessments, including coverage estimates [6]. A 2004 survey of IIS activity among 56 Centers for Disease Control and Prevention (CDC) grantees indicated that approximately 48% of U.S. children aged <6 years participated (i.e., had ≥ 2 vaccinations recorded) in IIS and that approximately 76% and 39% of public and private provider vaccination sites, respectively, had submitted data to an immunization information system during the last 6 months [5]. As IIS continue to improve nationally, they will likely be able to produce coverage estimates for all or small subgroups of children in a more timely fashion than other surveys. Moreover, well-developed IIS may eventually serve as the primary means to measure immunization coverage [4].

However, because IIS vary in their levels of provider and child participation and technological and operational capabilities, the use of these systems to monitor the overall impact of the CDC Recommended Childhood and Adolescent Immunization Schedule is limited. In 2004, the CDC funded state health departments in Arizona and Michigan to establish sentinel projects that were subsets of the state IIS to evaluate immunization programs in a specific population. Each sentinel project area was required to contain populations of at least 200,000 children aged ≤ 18 years in contiguous geographic regions; at least 50,000 of these children had to be <6 years of age, and ~95% of these children had to be participants in the IIS. In addition, sentinel projects were required to have ~95% of vaccine provider sites in the area as participants in the IIS. The IIS databases were also held to certain specifications; sentinel project databases had to a) have <5% of duplicate child records (ie, multiple records for the same child) and b) routinely collect vaccine manufacturer and vaccine lot number data. In addition, each site conducts quarterly, semi-annual, and annual monitoring of their IIS data. Based on these requirements, IIS sentinel site data in Arizona and Michigan are considered high-quality because they are population-based, comprehensive, and systematically evaluated.

Because 2004–05 was the first season that influenza vaccination was recommended among children aged 6–23 months, establishing an accurate baseline for the continued assessment of vaccination coverage in this age group was essential. During the 2004–05 season, however, an unexpected shortfall of influenza vaccine supply occurred. In response to this shortage, CDC recommended that vaccine be reserved for persons in certain priority groups, including children aged 6–23 months [7]. In this study, we describe influenza vaccination coverage among the newly recommended group of children aged 6–23 months in the 2 IIS sentinel projects during the 2004–05 influenza season.

## Methods

This study is based on data from 2 IIS sentinel projects in Arizona and Michigan through an agreement between CDC, the Arizona State Immunization Information System Project, and the Michigan Childhood Immunization Registry. In 2004, the AIIS sentinel project area included 7 primarily rural counties, with a population of ~61,000 children <6 years of age, of which 96% were IIS participants. Of the children in the sentinel site area, 50% were white, 32% were Native American or Alaskan Native, 17% were Hispanic, and 1% were black or Asian or Pacific Islander. Almost 20% of all persons included in the AIIS sentinel project area lived below the poverty level. An estimated 97% of provider sites in the sentinel project area participated in AIIS; among these, 74% were private provider sites and 26% were public provider sites.

In 2004, the MIIS sentinel site project area included 76 urban and rural Michigan counties with approximately 400,000 children <6 years of age, of which 93% were IIS participants. Of the children in the sentinel site area, 85% were white, 9% were black, 1% was Asian, and 5% was other or unknown. Less than 15% of persons included in the MIIS sentinel project area lived below the poverty level. An estimated 92% of providers in the sentinel project area participated in MIIS; among these, 89% were private provider sites and 11% were public provider sites.

Two measures of vaccination coverage are reported: a) receipt of ≥ 1 dose of influenza vaccine September 2004-March 2005 and b) receipt of ≥ 2 doses (i.e., fully vaccinated). Specifically, children were considered fully vaccinated if they had a) received no doses of influenza vaccination before September 1, 2004, but then received 2 doses September 1, 2004-March 31, 2005 or b) received ≥ 1 dose of influenza vaccine before September 1, 2004 and then received ≥ 1 additional dose September 1, 2004-March 31, 2005. Because children aged <6 months are not eligible for vaccination and because the recommendation at the time of the study calls for vaccination of children aged 6–23 months, analyses for both measures included only those children who were aged 6–23 months during the entire span of September 2004-March 2005 [8]. Thus, all children included in the study were born April 1, 2003-March 1, 2004 and had the same likelihood of being vaccinated during the time frame under study.

To assess possible differences in vaccine dose administration among children needing 1 and 2 doses in the 2004-05 season, we compared the dose administration distribution among children needing 1 dose (ie, children who had previously received a dose of influenza vaccine) and those needing 2 doses (ie, children who had never received a dose of influenza vaccine).

To further evaluate vaccination among children aged 6–23 months, we assessed influenza vaccination doses administered by public versus private sector provider sites. Public sector immunization provider sites included local health departments; federally qualified health centers (FQHC); Indian Health Service clinics and tribal clinics; community/migrant health centers/rural health clinics; military and other state and federal programs; women, infant, and children programs; public hospitals; and school district or school, daycare, and Head Start programs. Private sector immunization provider sites included private providers, faculty practices, health maintenance organizations, and private hospitals (not serving as an agent for an FQHC). In addition, vaccination coverage of ≥ 1 dose was assessed among children who were in the following 3 age cohorts as of September 1, 2004: a) children ~6 to 11 months of age, or the "youngest children"; b) children ~12 to 17 months of age, or the "middle children"; and c) children ~18 to 23 months of age, or the "oldest children". We also assessed the timeliness of influenza vaccine doses entered into AIIS and MIIS which were administered during the study time frame.All children aged 6–23 months participating in the IIS were included in this assessment and each sentinel project used state-specific procedures to extract data from the IIS and to ensure accurate vaccination record summary. Arizona used SQL Plus 8.0 (Oracle Corporation, Redwood City, CA), while Michigan used SAS for Windows 9.1 (SAS Institute Inc., Cary, NC, 2002) to create the datasets. The sentinel projects then sent their datasets to the CDC where analysis was conducted using Excel version 5.1 (Microsoft Excel, Redmond, Washington). The protocol was deemed to be exempt from the CDC institutional review board on the basis that data collected relate to programmatic evaluation as opposed to research involving human subjects.

## Results

From September 2004-March 2005, 10,161 children in AIIS and 65,578 children in MIIS met the age criteria for this study. Of the children in AIIS, 30% received ≥ 1 dose of influenza vaccination and 13% were fully-vaccinated; of the children in MIIS, 27% received ≥ 1 dose of influenza vaccine and 11% were fully-vaccinated.

**Figure 1:**
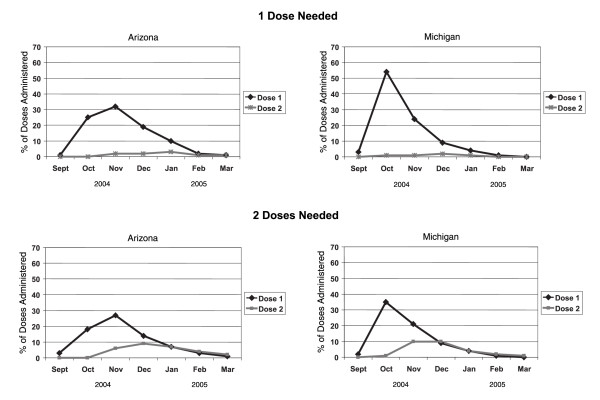
Percent of influenza vaccine dose administration among children needing 1 and 2 doses, 2004–2005 season.

During the same time period, 4,774 and 23,779 doses of influenza vaccine were administered to children aged 6–23 months in AIIS and MIIS respectively. In both sentinel projects, almost 70% of doses administered were dose 1 to children needing 2 doses and almost 30% were dose 2 administered to children needing 2 doses. Three percent and 5% of doses administered in AIIS and MIIS respectively were dose 1 to children needing 1 dose. Less than 1% of doses administered in both projects was dose 2 administered to children needing only 1 dose. Peaks in dose administration among children needing 1 and 2 doses were similar within project areas, with dose 1 peaking in November among both groups of children in AIIS and dose 1 peaking in October among both groups of children in MIIS (Figure 1).

**Figure 2:**
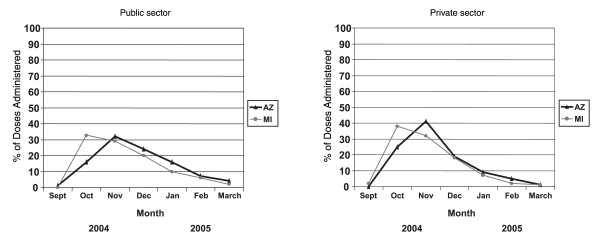
Public and private sector doses of influenza vaccine by sentinel project area, 2004–2005 season.

Variability was observed among doses administered in the public and private sector by state. In AIIS, public provider sites administered 61% of doses and private provider sites administered 39% of doses. In MIIS, public provider sites administered 26% of doses and private provider sites administered 74% of doses. The administration of all vaccine doses was highest for both public and private provider sites in October in Michigan and in November in Arizona (Figure 2).

In both sentinel projects, vaccination coverage with ≥ 1 dose was higher among the younger age cohorts. In the Arizona sentinel project area, vaccination coverage among children in both the youngest and middle age groups of children was 31%, and was 24% among the oldest group of children. In the Michigan sentinel project area, coverage was 29%, 28%, and 23% among the youngest, middle, and oldest age groups, respectively.

Of the ~5,000 vaccine doses administered to children aged 6–23 months in the Arizona sentinel project area, 60% were entered into AIIS within 2 weeks of administration and over 75% were entered within 30 days. Of the ~24,000 doses administered to children aged 6–23 months in the Michigan sentinel project area, 77% were entered into MIIS within 2 weeks of administration and over 85% were entered within 30 days.

## Discussion

Fall 2004 marks the first season in which ACIP recommended influenza vaccination of all children aged 6–23 months. Data from the Arizona and Michigan IIS sentinel projects indicated that almost 1/3 of children in this age group received ≥ 1 dose of vaccine and approximately 1/10 of these children were fully vaccinated during the 2004–05 season. Though approximately 30% coverage with ≥ 1 dose is not unexpected during the first season after a new recommendation, this coverage level, combined with the low coverage of fully vaccinated children illustrates that physicians and parents need to be targeted for educational efforts regarding the new recommendation. Several recent studies have added to the growing body of literature regarding the importance of a 2^nd ^dose of influenza vaccine in providing protection for young children. These studies have found minimal to no protection against influenza in young children without the 2^nd ^dose following the immunologic priming of the 1^st ^dose [9,10]. Indeed, a recent study indicated that during a year with suboptimal vaccine match no vaccine effectiveness was demonstrated in children aged 6–23 months after only 1 dose [11]. Such evidence regarding the importance of the 2^nd ^dose prompted ACIP to emphasize receipt of the booster dose in children in its 2006 influenza recommendations [12]. The vaccine administration distribution demonstrated in this study reveals that the distribution among children needing 1 and 2 doses of vaccine was similar in each project site. The overwhelming majority of doses were administered by the end of December, indicating that children who are receiving vaccine are receiving it before the typical peak influenza activity in the United States [13].

Whether and how much the shortage impacted coverage among children aged 6–23 months remains a question, difficult to answer during the first year of a recommendation for vaccination in this group. On the one hand, the vaccine shortage during the 2004–05 season was due to loss of vaccine manufactured by Chiron, a vaccine not licensed for use in children aged 6–23 months; thus, overall supplies for children <4 years of age were not affected and doses of vaccine for children remained unpurchased on the federal vaccine contracts at the end of the season. A survey of parents indicated that over 75% of parents who tried to get the vaccine for their children aged 6–23 months were able to do so [14]. In addition, anecdotal reports indicated a surplus of the pediatric formulation at the end of the season in public provider offices that used vaccine purchased through the federal contract (G. Wallace, CDC, personal communication, February, 2006). Although the Arizona Department of Health Services redistributed vaccine in response to the shortfall of doses from Chiron, we have no information about how this may have impacted the availability of vaccine for young children aged 6 – 23 months. Finally, despite the shortage, 100% of vaccine from Aventis Pastuer ordered by private-sector pediatric offices was shipped. However, it is still possible that the shortfall of vaccine could have impacted some children. Providers ordering only the Aventis Pasteur formulation, indicated for persons aged 6 months and older, may have gotten less vaccine than requested or may have used this product to vaccinate adults whom they had originally planned to vaccinate with the Chiron product, either of which could have decreased the amount of vaccine available in these practices for use in young children.

In both sentinel project areas, higher coverage levels were observed among the youngest and middle-aged groups of children compared with the oldest age group. The younger and middle age groups were within the recommended age range intervals for administration of other childhood vaccines during the entire study period, while the older age group was not. Therefore, the older age group may have had lower coverage because they were less likely to have a visit scheduled for administration of other vaccines.

Use of IIS provides a unique opportunity to assess immunization coverage rates. In theory, IIS can provide measures of vaccination coverage at all population levels [4]. Furthermore, unlike many surveys, IIS allow data to be collected in a manner which facilitates timely programmatic analysis and decision-making. The CDC IIS Technical Working Group established a goal for all IIS that called for immunization data to be received and processed by IIS within 30 days of vaccine administration [15]. In both sentinel project areas, over 75% of the influenza vaccine doses were entered into the IIS within 30 days after receipt of vaccine. In addition, the IIS enabled the prompt assessment of coverage for fully vaccinated children, including those receiving at least one dose of vaccine during the previous season.

The findings of this study are subject to at least 2 important limitations. First, though provider site participation rate in the IIS sentinel sites is high, the completeness of reporting by participating sites is unknown and there remain some sites in these areas who do not contribute their immunization data to the IIS. This lack of reporting could have resulted in an underestimate of vaccination coverage in the geographic area under study. Second, because study results are reported from only 2 sentinel project areas, they may not be generalizable to the overall U.S. population. However, the population included in these 2 project areas represents a demographically diverse group of individuals.

Other investigations have examined influenza vaccination coverage among children aged 6–23 months for the 2004–05 influenza season. A study conducted using the Behavioral Risk Factor Surveillance System (BRFSS), found coverage of ≥ 1 dose among U.S. children to be 48% September 2004 -January 2005 [16]. Similar to the current study, children included in the BRFSS study were aged 6–23 months during the entire season. A study conducted by Kaiser Permanente Northern California using cumulative coverage estimates September 2004 through March 2005 among children 6–23 months of age found receipt of ≥ 1 dose to be 58% [17]. However, comparability of these studies with the current study is limited because the populations under study are different and generalizability among them is difficult. For example, although we attempted to compare Arizona and Michigan state-specific BRFSS results to the results obtained in the IIS sentinel project study, the BRFSS sample size among children aged 6–23 months was too low to calculate a meaningful coverage estimate (Michael Link, CDC, personal communication, September 2005). In addition, unlike IIS data, BRFSS data are self-reported and subject to recall bias, potentially resulting in an overestimate of vaccination coverage [16]. Finally, estimates from the Kaiser study were obtained only from an insured, northern California population, which is likely demographically different from the 2 populations included in our study.

NIS influenza vaccination coverage estimates for the 2003–04 and 2004–05 seasons and the current study's data show increasing trends in coverage among children aged 6–23 months, which temporally parallels the strengthening ACIP vaccination recommendations [18,19]. Similar to the current study, childhood coverage assessments in children aged 6–23 months include only those children who were aged 6–23 months during the entire span under study. The 2003–04 NIS coverage estimates of ≥ 1 dose of vaccine in Arizona and Michigan were 16% and 17% respectively; and 7% and 8% respectively for children fully vaccinated. Estimates of 2004–05 NIS data and IIS data are similar, with NIS coverage of ≥ 1 dose in Arizona and Michigan at 27% [95% CI 21%, 33%] and 31% [95% CI 24%, 38%] respectively, and coverage of children fully vaccinated at 12% [95% CI 9%, 17%] and 16% [95% CI 11%, 22%] respectively [19].

## Conclusion

The current study indicates that almost 1/3 of children aged 6–23 months were vaccinated with ≥ 1 dose of influenza vaccination in 2 IIS sentinel project areas during the first year of the ACIP recommendation; however, full vaccination coverage was low. Much work is needed to fully implement the new recommendation and to increase coverage of the 2^nd ^dose of vaccine, including educating providers and parents on the importance of receipt of the 2^nd ^dose. Though effort needs to be placed in increasing provider site and child participation rates in IIS to help ensure that all influenza vaccine doses are entered into the IIS, this study illustrates that data from well-functioning IIS are useful for measuring vaccination coverage. In addition, IIS can produce more timely results than some other methods, which may not yield results until after the current influenza season and the data collection phase ends.

## Abbreviations

ACIP-Advisory Committee on Immunization Practices

AIIS-Arizona Immunization Information System

BRFSS-Behavioral Risk Factor Surveillance System

CI-Confidence Interval

CDC-Centers for Disease Control and Prevention

IIS-Immunization Information Systems

MIIS-Michigan Immunization Information System

NIS-National Immunization Survey

NHIS-National health Interview Survey

US-United States

## Competing interests

The author(s) declare that they have no competing interests.

## Authors' contributions

LAZ was responsible for the overall study design, instrument development, data collection and analysis, and manuscript preparation. DLB participated in the study design, instrument development, data analysis, and manuscript preparation. KSE and KG participated in data collection and analysis and manuscript preparation. WW participated in instrument development, data analysis, and manuscript preparation.

## Pre-publication history

The pre-publication history for this paper can be accessed here:


